# The Contribution of Small Vessel Disease to Neurodegeneration: Focus on Alzheimer’s Disease, Parkinson’s Disease and Multiple Sclerosis

**DOI:** 10.3390/ijms22094958

**Published:** 2021-05-07

**Authors:** Federico Paolini Paoletti, Simone Simoni, Lucilla Parnetti, Lorenzo Gaetani

**Affiliations:** Section of Neurology, Department of Medicine and Surgery, University of Perugia, 06132 Perugia, Italy; federico.paolinipaoletti@gmail.com (F.P.P.); simone.simoni@unipg.it (S.S.); lucilla.parnetti@unipg.it (L.P.)

**Keywords:** small vessel disease, neurodegeneration, neuroinflammation, Alzheimer’s disease, Parkinson’s disease, multiple sclerosis

## Abstract

Brain small vessel disease (SVD) refers to a variety of structural and functional changes affecting small arteries and micro vessels, and manifesting as white matter changes, microbleeds and lacunar infarcts. Growing evidence indicates that SVD might play a significant role in the neurobiology of central nervous system (CNS) neurodegenerative disorders, namely Alzheimer’s disease (AD) and Parkinson’s disease (PD), and neuroinflammatory diseases, such as multiple sclerosis (MS). These disorders share different pathophysiological pathways and molecular mechanisms (i.e., protein misfolding, derangement of cellular clearance systems, mitochondrial impairment and immune system activation) having neurodegeneration as biological outcome. In these diseases, the actual contribution of SVD to the clinical picture, and its impact on response to pharmacological treatments, is not known yet. Due to the high frequency of SVD in adult-aged patients, it is important to address this issue. In this review, we report preclinical and clinical data on the impact of SVD in AD, PD and MS, with the main aim of clarifying the predictability of SVD on clinical manifestations and treatment response.

## 1. Introduction

Brain small vessel disease (SVD) refers to a variety of structural and functional changes involving small perforating arterioles, capillaries and venules of the brain [1]. These changes are responsible for different lesions that can be seen on pathological examination or brain imaging, namely computed tomography (CT) and magnetic resonance imaging (MRI). Typical SVD lesions include white matter changes, lacunar infarcts and microbleeds. Superficial siderosis, perivascular spaces and small subcortical infarcts represent other possible manifestations [2]. Most of SVD is sporadic and associated with hypertension and other vascular risk factors, but a small proportion is related to genetic diseases, among which the cerebral autosomal dominant arteriopathy with subcortical infarcts and leukoencephalopathy (CADASIL) is the most frequent [3,4]. Brain SVD can be clinically silent, but either an increasing number or combinations of different lesion types might be associated with cognitive impairment, dementia, mood changes, gait disturbances and other movement disorders [5,6], often resembling clinical manifestations occurring in neurodegenerative and neuroinflammatory diseases. From a pathophysiological point of view, molecular mechanisms leading to SVD are interconnected with neurobiological pathways responsible for neurodegeneration and neuroinflammation, which typically include protein misfolding, derangement of cellular clearance systems, mitochondrial impairment and immune system activation [7]. From a clinical perspective, the exact role of SVD in common neurological diseases such as Alzheimer’s disease (AD), Parkinson’s disease (PD) and multiple sclerosis (MS), and its impact on response to pharmacological treatments, is still a matter of debate. Since SVD is a common finding in adult-aged life, when it also can co-occur with central nervous system (CNS) degenerative or inflammatory diseases, it is relevant to address such issues. Recently, the Joint Program for Neurodegenerative Disease (JPND) promoted an initiative coordinated by 55 international experts who surveyed and assessed the currently available data on this topic, in order to improve the understanding of how vascular disease affects brain structure and function, and to address methodological issues for cerebrovascular disease in neurodegeneration research [8]. In this review, we provided an overview about the molecular mechanisms and pathophysiological aspects linking SVD and degenerative/inflammatory processes occurring in the CNS. Therefore, we considered AD, PD and MS, focusing on the potential role and the clinical impact of SVD in these common neurological disorders, also highlighting the possibility to specifically measure SVD with fluid biomarkers and to target it with disease-modifying therapies.

## 2. Common Molecular Mechanisms and Pathophysiological Aspects of Small Vessel Disease and Neurodegeneration

**Figure 1:**
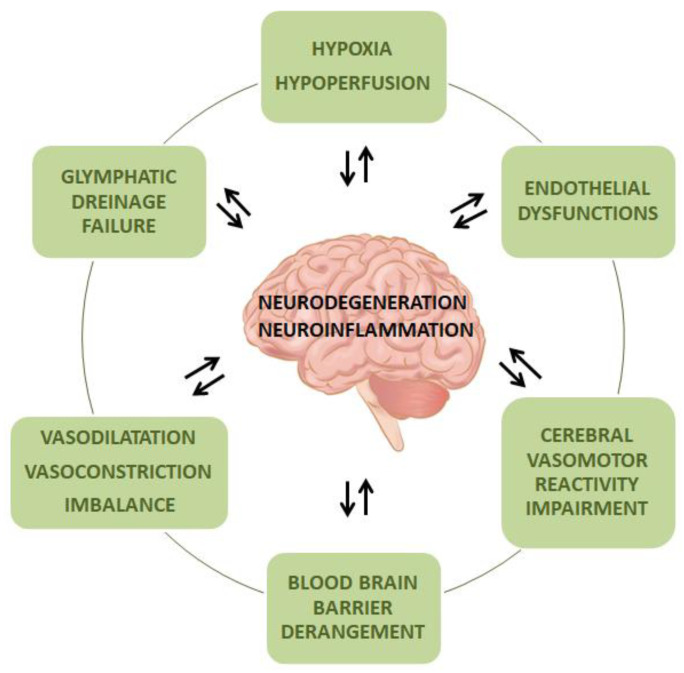
The figure shows the main pathophysiological mechanisms that link neurodegeneration/neuroinflammation and small vessel disease.

Neurodegenerative diseases like AD, PD, atypical parkinsonisms, frontotemporal dementia (FTD) and amyotrophic lateral sclerosis (ALS) show substantial differences in terms of clinical manifestations, while share pathophysiological features such as protein misfolding, prion-like propagation and progressive accumulation of proteinaceous aggregates [9]. Multiple SVD-related mechanisms are involved in the processes underlying neurodegeneration (Figure 1), but specific knowledge is still lacking for most of them.

Hypoxia represents one of the most investigated aspects, probably due to its easy reproducibility also in animal models where hypoperfusion can be obtained through the stenosis of common carotid arteries, by applying microcoils or constrictor devices. Hypoxia seems to enhance both amyloidosis and tauopathy, which are the two hallmarks of AD-related pathology [10,11]. Hypoxia-inducible factor 1α (HIF 1α) increases the transcription of β-secretase and γ-secretase, which are involved in the amyloidogenic cascade [12]. Additionally, hypoxia downregulates neprilysin, an Aβ degrading enzyme [13]. In rodents, ischemia leads to neuronal accumulation of hyperphosphorylated tau, by mechanisms involving the mitogen-activated protein kinase (MAPK) [14], with subsequent formation of abnormal tau filaments resembling those present in human tauopathies [11]. In a recent experiment with mice undergoing transient middle cerebral artery occlusion, a comprehensive proteomic analysis of post-ischemic aggregated proteins was performed. RNA-binding proteins, such as TAR DNA-binding protein 43 (TDP-43) and fused-in-sarcoma (FUS) associated with FTD and ALS, were found to be the largest group of proteins susceptible to aggregation during ischemia, suggesting ischemia as a trigger condition for protein misfolding and aggregation in neurodegenerative disorders [15]. Furthermore, hypoxia plays an important role also in MS demyelinating lesions. While pattern I and II lesions (i.e., lesions with prominent T cells and macrophages infiltration and antibody and complement deposition) have morphological characteristics consistent with an autoimmune etiology, pattern III lesions show distal oligodendrogliopathy with dysregulated myelin protein expression and oligodendrocyte apoptosis, with features suggesting a central role for hypoxia, based on the similarity with demyelination in the penumbra around an ischemic focus [16].

Independent of Aβ, tau and other misfolded proteins deposition, hypoxia causes mitochondrial machinery derangement, which is a pivotal event in both inflammatory and degenerative CNS disorders [17], suppresses glutamate reuptake by astrocytes, resulting in glutamate-mediated toxicity [18], and alters expression of vascular-specific genes, which causes endothelial dysfunction [19]. Endothelial dysfunction plays a key role in the interconnected links between SVD and neurodegeneration. In the CNS, endothelium functions include vascular tone and blood flow regulation, protection against thrombosis, inflammation and fibrosis, and resident immune system modulation, molecules transport across the blood–brain barrier (BBB), and cytotoxic products clearance. Endothelial-related signaling is mediated by a series of vasoactive agents including nitric oxide (NO), which is also involved in mitochondrial machinery, synaptic transmission, neuronal and glial homeostasis [19]. Endothelial dysfunction causes decreased NO availability, which in turn is responsible for increased expression of amyloid precursor protein (AβPP) and AβPP cleaving enzyme 1 (BACE-1) leading to Aβ increase [20]. In PD, both experimental and clinical data suggest endothelial involvement. Capillary loss, abnormal fragmented capillaries and endothelial fenestrations can be detected in substantia nigra, brainstem and cerebral cortex in brain samples deriving from both MPTP-treated animal models and PD patients, where a compensatory formation of immature and leaky vessels is also evident [21,22].

Endothelial dysfunction is also responsible for cerebral vasomotor reactivity (CVR) impairment. CVR represents the basis for cerebral blood flow (CBF) regulation according to the brain metabolic status and is usually defined as the amount of change in CBF in response to a vasodilating stimulus. Indeed, in clinical settings, CVR can be determined by measuring CBF changes following a vasoactive challenge (i.e., CO_2_ or acetazolamide administration). CBF changes can be calculated directly by neuroimaging (functional magnetic resonance imaging or nuclear imaging) or indirectly through the measurement of blood flow velocity in major cerebral arteries by transcranial sonography [23]. Clinical research showed that CVR derangement is present in AD, in which it is also associated with an increased risk of progression from mild cognitive impairment (MCI) to dementia [24,25]. There is also evidence of similar dysfunctions in PD [26] and MS [27]. From a molecular point of view, it should be considered that amyloid deposition involves not only brain parenchyma but also cerebral vessels walls, directly constricting cerebral arteries and hampering CVR [28]. Additionally, these deposits compromise the ability of endothelial cells to produce vasodilators like NO, thus impairing CVR and other mechanisms, which regulate CBF such as autoregulation and neurovascular coupling [29]. Cholinergic projections originating from the Meynert nucleus can induce vasodilation both directly through the acetylcholine release and indirectly through the stimulation of brainstem NO-releasing interneurons. Thus, cholinergic transmission dysfunction, invariably present in AD, but also evidenced in PD and MS, represents another mechanism leading to CVR failure [30,31,32]. In both neurodegenerative and neuroinflammatory disorders, reactive astrocytes (hypertrophied astrocytes overexpressing glial fibrillary acid protein) are supposed to contribute to CVR impairment by producing increased levels of endothelin-1 (ET-1) with vasoconstrictor properties [33,34]. ET-1 expressing astrocytes have been found in AD and MS patients [34,35], and both CSF and serum ET-1 levels are increased in MS [36,37]. Particularly, in the CSF, ET-1 levels were higher in patients with relapsing remitting MS during an acute phase than in clinically stable patients [37].

Endothelial dysfunctions impact negatively on the so-called neurovascular unit, which consists of different cell types (endothelial cells, pericytes, vascular smooth muscle cells, glial cells and neurons) and represents the functional basis for BBB integrity. As BBB is altered, Aβ drainage from the perivascular space is reduced, thus facilitating its accumulation within the brain [38]. Brain Aβ homeostasis is also regulated via interaction with two main receptors expressed by the endothelial cells. The lipoprotein receptor-related protein 1 (LRP-1) is responsible for Aβ clearance from the brain while the receptor for advanced glycation end products (RAGE) mediates Aβ re-entry into the brain from circulation. In AD, there is evidence of upregulation of influx receptor RAGE and downregulation of efflux receptor LRP-1 [39]. ApoE is associated with Aβ in the perivascular space and this interaction seems to be relevant for perivascular drainage of amyloid peptides. APOE genotype can affect the efficiency of this drainage pathway, through differences in Aβ–apoE interactions [40], also enhancing endothelial dysfunction. In SVD, apoE has been detected in vessel wall lesions [41] and its ε4 allele, which is well-known as the major genetic risk factor for sporadic AD [42], is also associated with the severity of SVD [43]. Endothelial dysfunctions might be also associated with the impairment of glymphatic pathway, a fluid clearance system recently identified in rodent brain, which clears toxic solutes from brain into meningeal and cervical lymphatic drainage vessels, throughout perivascular and perineural spaces, facilitated by aquaporin-4 (AQP-4) water channels. Glymphatic dysfunctions have been demonstrated in animal models of AD and SVD, most likely in relation to perturbed expression of AQP-4 [44].

## 3. Role of Small Vessel Disease in Alzheimer’s Disease

AD is the most frequent neurodegenerative disorder evolving to dementia [45]. Cerebrovascular diseases are considered the second most common cause of dementia in European and American countries. Among elderly, there is evidence of co-occurrence of AD and SVD, which share several risk factors, namely hypertension in midlife, diabetes mellitus, dyslipidemia, high levels of plasmatic homocysteine and ε4 allele [46]. Mixed AD/SVD pathology is frequently observed in AD patients, and this trend increases with age [47]. However, the relative contribution of each pathology to cognitive impairment needs to be better elucidated.

One of the most interesting aspects is to understand whether SVD plays a role in the Alzheimer’s continuum, by promoting the transition from preclinical phase to prodromal stage, and from MCI to dementia. In a recent longitudinal investigation focusing on preclinical AD, the authors found a significant association between baseline white matter hyperintensity (WMH) volume and time to symptom onset, with MCI appearance within 6 years among patients with low cerebrospinal fluid (CSF) t-tau levels, but later among those with high t-tau [48]. SVD (as reflected by WMH volumes) increased the risk of progression to MCI when levels of neurodegeneration (as reflected by t-tau concentration) are low, which argues against a synergistic effect between SVD and neurodegeneration on cognitive decline and supports the idea that t-tau reflects amyloid-dependent neuronal damage while other causes of neurodegeneration, i.e., SVD, are not encompassed. Otherwise, no interactions between baseline WMH volume and CSF Aβ42 and p-tau levels were found, with respect to the time to symptom onset [48], suggesting that cerebral SVD and AD pathologies have substantially independent effects on the risk of progression to MCI. Different studies investigated the contribution of SVD to the risk of progression from MCI to dementia in AD, often showing conflicting results. Some authors showed a relationship between white matter changes and progression to dementia in MCI patients [49,50], while others failed to find any association between white matter changes and cognitive decline [51,52]. In the ADNI (Alzheimer disease neuroimaging initiative) study, a higher WMH volume at baseline and at 1-year follow-up was associated with greater cognitive impairment [53]. In 161 pathologically confirmed AD individuals from the OPTIMA (Oxford Project to Investigate Memory and Aging) investigation, no correlations were found between cerebral subcortical SVD severity and cognitive scores collected by means of MMSE (mini mental state examination) and CAMCOG (Cambridge cognition examination) during the last two years of patients’ life. On the contrary, SVD severity was significantly related to age and was slightly, but significantly, higher in females than in males [54].

Another open issue relies on the characteristics that make AD patients more likely to have an underlying SVD pathology. With respect to onset age, late-onset patients (age at onset >70 years old) were shown to exhibit a greater amount of SVD, in terms of both WMH and lacunar infarcts, compared to early-onset patients (age at onset <60 years old) [55]. Interestingly, the association between late-onset AD (LOAD) and SVD seems not to be mediated by a genetic predisposition (ε4 allele and AD family history) to AD. Indeed, in a cross-sectional analysis of cognitively unimpaired subjects aged 40–59 years recruited from the PREVENT dementia study, the authors found no relationship between ε4 carrier status or history of parental dementia and SVD as represented by WMH volume and microbleeds [56]. On the contrary, in cognitively unimpaired individuals (mean age of 58 years old), WMH distribution was associated with ε2 allele [57]. Additionally, in patients with MCI and dementia due to AD, ε2 carriers showed larger WMH volumes compared to ε4 carriers and greater prevalence of microbleeds compared to ε3 homozygotes [58]. Though ε2-carrier status is classically considered a protective factor for AD and it is not a common finding in AD patients, ε2 carriers with AD showed peculiar characteristics, i.e., greater SVD-related burden, more pronounced non-amnestic impairments, and different atrophy pattern with asymmetric distribution (left > right) and relative medial temporal structures sparing, with respect to ε4 carriers and ε3 homozygotes [58]. Recently, Ferreira et al. [59] investigated SVD prevalence and distribution in AD patients, which were categorized in four different subtypes according to brain atrophy pattern on MRI: (i) typical (with balanced atrophy in the hippocampus and associative cortex), (ii) limbic-predominant (with prevalent atrophy in the hippocampus), (iii) hippocampal-sparing (with predominant atrophy in the associative cortex) and (iv) minimal atrophy. Both WMH and microbleeds showed the highest prevalence in limbic-predominant AD, with the lowest representation in minimal atrophy subtype. With respect to SVD distribution, all subtypes showed more pronounced WMH in frontal and posterior brain regions. Otherwise, microbleeds topography differed across subtypes, with typical and limbic-predominant AD having higher prevalence in all lobar regions, hippocampal-sparing AD showing higher prevalence in posterior lobes, cerebellum and deep regions and minimal atrophy AD having higher prevalence only in posterior lobes and cerebellum.

## 4. Clinical Impact of Small Vessel Disease in Parkinson’s Disease

The hypothesis that vascular changes in the brain may play a role in parkinsonism has long been debated. When considering SVD and parkinsonism, a first major distinction should be made between idiopathic PD, a neurodegenerative disease during which ischemic cerebrovascular lesions can occur, and the so-called vascular parkinsonism (VP), which has a predominantly cerebrovascular etiology. VP is limited to a condition where vascular lesions in the substantia nigra (SN) or nigro-striatal pathway lead to an asymmetric parkinsonism, possibly presenting with presynaptic dopamine transporter (DAT) deficiency [60]. Nevertheless, the term VP is commonly applied to refer to ‘lower-body’ parkinsonism with gait and posture disturbances, in association with white matter disease [61]. Given the nebulous diagnostic borders, the prevalence of VP varies widely, ranging from 2% to 29% of all cases of parkinsonism, depending on population and criteria [60]. Additionally, clinico-pathological investigations have shown heterogeneity in the clinical presentation of VP, often overlapping with PD [62,63].

The presence of brain vascular lesions in the course of idiopathic PD may be found in almost 25% of PD patients, although their contribution to the clinical picture need to be better elucidated [63,64,65,66]. Clinical, imaging and pathological studies suggested a negative impact of cerebrovascular disease and vascular risk factors including diabetes, hypertension and dyslipidemia, on both cognition and motor tasks, particularly affecting posture and gait among the latter [67,68]. Whilst α-synuclein-related neurodegeneration and brain amyloidosis are known to be responsible for the progression of cognitive impairment in PD, cerebral SVD burden has been shown to be a contributing factor [69,70,71]. Even subclinical cerebrovascular disease has been linked to greater motor severity and gait impairment in PD patients [71,72,73]. Additionally, a significant association was found between disease severity, as expressed by the Hoehn and Yahr stage, and cerebral vascular lesions, particularly those located in peri-ventricular and hemispheric white matter or infratentorial areas (i.e., brainstem and/or cerebellum) [66].

Specific disease phenotypes and motor manifestations in PD seem to be more vulnerable to cerebrovascular damage than others. Referring to white matter cerebrovascular burden, axial impairment was shown to worsen more than bradykinesia, while no relationship was observed with either tremor or rigidity [68]. Cerebral SVD burden has shown to be related to impaired gait/posture in patients with PD, independent of age, hypertension, previous history of ischemic stroke, LDL and blood cholesterol level and MMSE and MoCA scores [68,74,75]. A multicenter study has recently confirmed that SVD is associated with increased severity of parkinsonism, also negatively affecting the response to chronic levodopa treatment. Overlapping syndromes between PD and cerebrovascular disease may create mixed motor phenotypes, which can explain the limited responsiveness of some of the motor and cognitive features to anti-parkinsonian therapies [68].

A few patients presenting progressive reduction in amplitude and speed in rapid repetitive movements, thus fulfilling diagnosis of possible PD, show a normal FP-CIT scan and have no benefit from levodopa treatment. These cases are generally characterized by the presence of gait impairment, which is defined as frontal, magnetic or “apraxic”, also including varying degrees of slowed and short-stepped gait with freezing. This condition should be more likely attributed to a vascular pseudo-parkinsonian state, being an expression of cognitive rather than primarily motor impairment, which constitutes another possible phenotypes in addition to idiopathic PD and VP [60].

## 5. Small Vessel Disease as A Potential Contributor to Neurodegeneration in Multiple Sclerosis

MS is a chronic disease with a young adulthood onset and an extremely variable course [76]. Part of this variability is linked to inflammation-related aspects, according to which patients may have a variable degree of immune activation within the CNS, with a subsequent variable course of the disease [77]. Besides this, however, a paradox exists between inflammatory biomarkers severity and disability, with patients having a low evidence of inflammation according to brain MRI biomarkers, and a fast progression of disability [78]. Such clinical-radiological paradox may be due to the contribution, in the disease severity, of mechanisms other than inflammatory ones. SVD has been advocated as a possible contributor to such variability. It is for instance well known that MS patients with vascular comorbidities (e.g., diabetes, hypertension and heart diseases) require walking aid sooner as compared to those without these diseases [79]. The effect of SVD on determining a different disease course may be due to a booster effect on the neurodegeneration taking place along the disease course. Indeed, MS patients with vascular diseases have, on average, lower brain volumes compared to those without these comorbidities [80].

Post-mortem studies have demonstrated that in MS brains, vascular changes beyond the characteristic perivenular inflammation are present, affecting the arterioles even in the absence of vascular comorbidities [81]. In MS brains, indeed, arteriolar pathology is significant, with large periarteriolar spaces and more severe periarteriolar inflammation and hemosiderin deposition having been found also outside inflammatory lesions [81]. This evidence suggests that MS pathology may have an influence on the entire vascular tree, thus enhancing the possibility of SVD development [81]. In turn, periarteriolar inflammation may contribute to the cerebral blood flow changes that have been described in normal-appearing white matter since the earliest stages of MS [82]. Indeed, in MS a diffused cerebral hypoperfusion has been documented and associated to chronic hypoxia, focal lesion formation, diffuse axonal degeneration and to clinical outcomes, namely cognitive dysfunction, and fatigue, which are important determinants of disability due to MS [82]. Therefore, an accelerated SVD in MS may contribute to a hypoxic milieu that enhances brain vulnerability to neuronal degeneration [83].

The link between SVD and neurodegeneration may fill the gap between inflammation and MS disability progression, which are frequently apparently dissociated. The contribution of SVD into MS pathophysiology is certainly relevant to clinical practice. Indeed, vascular risk factors may require a more aggressive monitoring and treatment in MS patients compared to non-MS subjects [81].

## 6. Small Vessel Disease in the Spectrum of Frontotemporal Lobar Degeneration

Frontotemporal lobar degeneration (FTLD) represents a heterogeneous group of neurodegenerative disorders that are characterized by aggregates of different proteins such as TDP-43 and FUS. The spectrum of clinical manifestations includes dementia, variably associated with behavioral changes, primary progressive aphasia, parkinsonism and motor neuron-related phenotypes [84]. In turn, FTLD subtypes characterized by tau pathology (FTLD-tau) include Pick disease (PiD), argyrophilic grain disease (AGD), progressive supranuclear paralysis (PSP) and corticobasal degeneration (CBD). Hypoxia was demonstrated to be a triggering factor for protein misfolding and aggregation even in cases of non AD-related tauopathy, thus begging the question whether SVD plays a similar role in all FTLD-subtypes. As found in a post-mortem investigation, PiD showed higher SVD burden compared to other FTLD-tau subtypes, also revealing a colocalization between SVD-like lesions and tau-related demyelination areas in the frontal and temporal cortex. Additionally, PiD cases were the youngest in terms of disease onset, suggesting that SVD may cause earlier clinical onset in patients with an underlying PiD-related pathology. However, similar degrees of SVD were not necessarily associated with PiD-related demyelination in control brains from older individuals [85]. This finding is consistent with the idea that SVD is not necessary for FTLD-tau, rather it is triggered by severe cortical tau pathology in PiD, where it might be a contributing factor for the clinical manifestation.

## 7. Biomarkers for Small Vessel Disease: Beyond Neuroimaging

The most evident manifestations of SVD are those observable through brain imaging particularly brain MRI [86]. Imaging fingerprints of SVD include lacunar infarcts, white matter changes, perivascular spaces and microbleeds. In 2013, an international working group provided a set of recommendations to standardize images acquisition, reporting and interpreting for SVD and summarized the standards for reporting vascular changes on neuroimaging (STRIVE) in a position paper [2]. SVD-related neuroradiological findings can be either silent, without prominent symptoms, or associated with clinical manifestations ranging from cognitive decline to motor disturbances, resembling those occurring in neurodegenerative diseases. Thus, given the coexistence of SVD with neurodegenerative processes, brain imaging alone is not able to establish if SVD is sufficient to explain the clinical picture or, instead, if a neurodegenerative origin should be considered. Furthermore, imaging findings are evident when the vascular disease is too advanced and extensive. They represent the final picture of different cerebrovascular pathologies, failing to catch early and different molecular mechanisms underlying SVD. CSF surrounds the CNS and provides a source of biomarkers, which can mirror the pathophysiological processes taking place within the brain. Among neurological diseases, AD offers the best example of the usefulness of CSF biomarkers in clinical practice. CSF biomarkers reflecting the coexistence of amyloidosis and tauopathy, Aβ42/Aβ40 ratio and phosphorylated tau at threonine 181 (p-tau) respectively, allow one to claim the presence of AD-related neurobiology, independent of disease stage and clinical presentation [45]. Nevertheless, these biomarkers make it possible to detect or rule out an AD signature, without identifying different neurodegenerative origins or SVD involvement. There is also evidence for a characteristic CSF signature in patients with SVD (Table 1). Among the molecules that can be investigated in the CSF, those linked to oligodendrocyte-derived myelin sheath like myelin lipid sulfatide and myelin basic protein (MBP) seem to be some of the most promising biochemical markers for SVD. Increased levels of CSF sulfatide and MBP have been related to manifest SVD, also distinguishing it from other neurologic conditions like AD and chronic hydrocephalus [87,88,89]. The relationship between these biomarkers and SVD may reflect the remyelinating response to demyelination, a process that seems to take place before the axon is affected. Neurofilament light chain (NfL), a marker of axonal damage, was found to increase early in the CSF in SVD, even in non-disabled patients with white matter changes, and in MCI patients who later developed vascular dementia [90,91]. In AD, while CSF t-tau reflects amyloid-dependent neurodegeneration or increased tau secretion from amyloid-affected neurons, CSF NfL is considered a measure of both amyloid-dependent and independent neuronal loss [92]. Thus, higher CSF NfL levels might be expected in cases of mixed pathology, i.e., AD-related pathology associated with SVD, compared to pure AD-pathology. The role of NfL in dissecting the contribution of amyloidosis and SVD in neurodegeneration may certainly be relevant for clinical practice and should be investigated also in other neurodegenerative diseases. For instance, in PD, it should be investigated whether CSF NfL correlates with SVD, thus discriminating the amount of neuronal loss due to SVD from that due to other characterizing PD pathophysiological mechanisms (e.g., synucleinopathy, mitochondrial and lysosomal dysfunctions). Unfortunately, in MS CSF NfL changes are expected to reflect white matter axonal damage, either due to inflammatory demyelination or to SVD [92]. Thus, other CSF biomarkers more linked to SVD should be investigated.

A clue in the search for CSF SVD biomarkers comes from studies on patients with SVD-related dementia (both pure SVD and mixed SVD/AD pathology), who showed increased levels of CSF α-1 antitrypsin, tissue inhibitor of metalloproteinase-1 (TIMP-1), plasminogen activator inhibitor-1 (PAI-1) and apolipoprotein H (ApoH), with respect to pure AD patients and healthy control subjects [93]. The best discrimination between SVD and AD was obtained by combining a panel of different biomarkers reflecting AD neurobiology (Aβ42, p-tau and t-tau), axonal damage (NfL), demyelination (MBP) and matrix remodeling pathway (TIMP-1 and matrix metalloproteinases) [89].

Further studies involving larger and longitudinal cohorts are needed to better understand the role of fluid biomarkers in SVD. However, these preliminary findings seem to indicate that there might be a CSF signature for SVD. Compared to brain imaging, CSF biomarkers seem to provide a more precise measurement of SVD contribution to the clinical picture when neurodegenerative processes coexist with SVD. A CSF biomarkers-based approach could lead to the quantification of SVD in neurological diseases and, therefore, to its identification as a possible therapeutic target.

## 8. SVD as a Target for Possible Therapeutic Approaches

SVD is a common accompaniment of ageing and frequently coexists with neurodegenerative diseases. The interaction between vascular risk factors, brain SVD and CNS diseases may lead to an acceleration of the neurodegenerative process itself. Reducing the direct effects of ischemia-related neuronal loss would be a key approach to limit the damaging effects from comorbid cerebrovascular disease on both motor and cognitive manifestations of neurological diseases. Thus, treatments preventing vascular degeneration in the early phases of such diseases may improve vascular remodeling in the brain, providing new targets to ameliorate motor and cognitive symptoms burden in neurodegenerative and neuroinflammatory disorders. Nevertheless, the role of SVD seems to be underestimated when potential disease-modifying strategies are considered in the field of neurodegenerative disorders, which could explain, at least partially, clinical trials failure with some neuroprotective agents. Indeed, most therapeutic efforts against neurodegeneration have focused so far on the development of the so-called single-action agents, which directly target only neuronal cells, by reversing neuronal dysfunction and/or or protecting neurons from specific insults. Most preclinical and clinical studies have shown that such drugs are unable to modify the disease course. In the last few years, the vascular-neuronal-inflammatory model has been proposed, to indicate that vascular damage, neurodegeneration and neuroinflammation comprise a pathophysiological triad, which occurs in multiple neurological disorders. Accordingly, it is conceivable that multiple-action agents, compared to one-action agents, could have a better chance in controlling the complex disease mechanisms underlying neurodegeneration [94]. In this scenario, a series of molecules can be considered as potential targets for disease-modifying drugs. NO is a paradigmatic example for the vascular-neuronal-inflammatory model. Its depletion contributes to capillary constriction and microcirculation impairment, and impacts negatively on synaptic transmission, neuronal survival and glial homeostasis. NO inhalation and nitrite infusion have been suggested to improve microcirculation and tissue oxygenation in different models of neurovascular diseases [95,96,97]. Similar beneficial effects are supposed for neurodegenerative diseases [98]. Though promising, NO is a two-edge sword. On the one hand, a decreased NO availability is deleterious for microcirculation, neuronal and glial functions. On the other hand, excessive NO is one of most important mediators of excitotoxicity [98]. Given these premises, it is challenging to concentrate NO in specific brain regions and precise NO amount is difficult to maintain.

Similarly, the vascular endothelial growth factor (VEGF) and other angioneurins may have multiple actions in the CNS. Local intracerebral implantation of VEGF-secreting cells was shown to enhance vascular repair, reduce amyloid burden and improve learning and memory in a mouse model of AD [99]. Studies on ALS animal models showed that intracerebroventricular or intramuscular administration of a VEGF-expressing lentiviral vector promote angiogenesis, increase blood flow through the spinal cord and exert direct protective effects on neuronal cells, thus reducing disease-specific pathology and extending survival [100,101]. Given these preclinical results, a phase I–II clinical trial was carried out to evaluate safety and tolerability of intracerebroventricular VEGF infusion in patients with ALS (unpublished results, clinicaltrial.gov: NCT00800501).

Research focusing on vascular-neuronal-inflammatory model is promising, having the potentiality to change our view about degenerative and inflammatory neurological diseases. Further insights are needed, leading to the development of new neurovascular therapies.

## 9. Concluding Remarks

In adult-advanced age SVD often co-occurs with neurodegenerative or neuroinflammatory diseases. At the molecular level, hypoperfusion and hypoxia may be triggering factors for neurobiological pathways leading to neurodegeneration, i.e., protein misfolding and proteinaceous aggregates deposition. Such molecular mechanisms, in turn, promote endothelial dysfunction, CVR impairment, BBB breakdown and glymphatic pathway derangement, in a vicious cycle that further amplifies both hypoperfusion and neurodegeneration/neuroinflammation. In front of common molecular bases and neurobiological mechanisms, CNS diseases such as AD, PD and MS show different clinical phenotypes, disease progression and outcomes, and can benefit from different treatment approaches, according to their occurring either as a single entity or as mixed pathology combined with SVD. In AD, the co-occurrence of SVD may cause earlier clinical onset, also fastening cognitive decline. Similarly, cerebrovascular disease is a contributing factor for both cognitive impairment and motor progression in PD, in which axial manifestations such as posture alterations and gait disturbances are the most influenced by the presence of SVD. In MS, SVD seems to be responsible for earlier occurrence of disability, partially filling the paradoxical gap between inflammation and disease progression. In front of a well-established prognostic role of SVD in these common neurological diseases, the relationship between SVD and its clinical characteristics is still unclear. SVD can be either clinically silent or manifest similarly to neurodegenerative and neuroinflammatory diseases, in absence of a specific correlation between clinical features and imaging findings. In the presence of neuroimaging evidence of SVD, a possible degenerative or inflammatory origin of the clinical picture should be always ruled out. In this scenario, biomarkers reflecting amyloidosis, tauopathy, neurodegeneration and intrathecal inflammation, already used in routine clinical practice in neurodegenerative/neuroinflammatory disorders, could be integrated with novel and promising biomarkers of vascular remodeling and demyelination/re-myelination processes. In research settings such as clinical trials for disease-modifying therapies, the application of CSF biomarkers measuring the contribution of SVD in the context of neurological diseases is also valuable, in order to select more homogeneous cohorts, verify target engagement and monitor diseases outcomes. Research focusing on the vascular-neuronal-inflammatory model seem to be promising, and the subsequent development of multiple-action therapies strengthens again the need of having a panel of biomarkers reflecting the links between SVD, neurodegeneration and neuroinflammation.

## Figures and Tables

**Table 1:** The table shows the main findings on cerebrospinal fluid biomarkers to investigate small vessel disease within neurological diseases. Legend. AAT: α-1 antitrypsin; AD: Alzheimer’s disease; ApoH: apolipoprotein H; CSF: cerebrospinal fluid; HC: healthy controls; MBP: myelin basic protein; MCI: mild cognitive impairment; MD: mixed dementia (AD + SVD); NfL: neurofilament light chain; NPH: normal pressure hydrocephalus; PAI-1: plasminogen activator inhibitor 1; SVD: small vessel disease; TIMP-1: tissue inhibitor of metalloproteinase 1; VaD: vascular dementia; WML: white matter lesions, ↑: increase.

Pathophysiological Mechanisms	CSF Biomarkers	Patients	Main Findings
Demyelination and re-myelination processes	sulfatide	VaD vs. AD and HC VaD vs. NPH	↑ in VaD ↑ in VaD
	MBP	VaD vs. HC	↑ in VaD
Axonal damage	NfL	SVD	↑ in SVD ↑ in MCI, predicting VaD
Vascular remodeling	AAT	SVD and MD vs. AD	↑ in SVD and MD
	PAI-1	SVD and MD vs. AD	↑ in SVD and MD
	TIMP-1	SVD and MD vs. AD	↑ in SVD and MD
	ApoH	SVD and MD vs. AD	↑ in SVD and MD
